# Effect of liuzijue qigong on patients with stable chronic obstructive pulmonary disease: A systematic review and meta-analysis

**DOI:** 10.1097/MD.0000000000027344

**Published:** 2021-10-15

**Authors:** Pincao Gao, Fang Tang, Weiguo Liu, Kai He, Yu Mo

**Affiliations:** a College of Physical Education and Health, Guangxi Normal University, No. 1 Yanzhong Road, Yanshan District, Guilin, Guangxi, People's Republic of China; b College of Rehabilitation and Health, Hunan University of Medicine, No. 492 Jinxi South Road, Huaihua, Hunan, People's Republic of China; c Hunan Provincial Key Laboratory of Dong Medicine, Hunan University of Medicine, Huaihua, PR China.

**Keywords:** chronic obstructive pulmonary disease, dyspnea, liuzijue qigong, lung function

## Abstract

**Backgrounds:**

Chronic obstructive pulmonary disease (COPD) is a common, preventable disease of airflow limitation that accounts for the third leading deaths of any disease process in the worldwide. Health benefits of liuzijue qigong (LQG) on patients with stable COPD has been assessed. This study was designed to perform a systemic review and meta-analysis of the effect of Liuzijue breathing exercise on patients with stable COPD.

**Methods:**

Published articles from 1970 to December 2020 were conducted using electronic searches. Two independents reviewers conducted data extraction. The Cochrane risk of bias assessment tool was used to evaluate the quality of the included studies.

**Results:**

A total of 16 eligible trials with 1039 patients with stable COPD were identified. Compared with control group, the pool meta-analysis of LQG showed a significant improvement in forced expiratory volume in one second (FEV1) (MD = −0.16, 95% CI [0.09, 0.23], *P* < .00001), FEV1% (MD = 9.71, 95% CI [8.44, 10.98], *P* < .00001), the ratio of forced expiratory volume to forced vital capacity in the first second (FEV1/FVC [%]) (MD = 4.81, 95% CI [2.12, 7.51], *P* = .0005), 6 minutes walking distance (6MWD) (MD = 21.89, 95% CI [14.67, 29.11], *P* < .00001), health-related quality of life (SMD = −0.84, 95% CI [−1.12,-0.55], *P* < .00001) and modified medical research council dyspnea scale (mMRC) (MD = −0.73, 95% CI [−0.96, −0.50], *P* < .00001). The observed effect was more pronounced for short term and medium-term duration interventions of study. It also showed improvements in the secondary outcome measures by LQG.

**Conclusions:**

In this systematic review and meta-analysis, LQG can improve lung ventilation function, exercise endurance and health-related quality of life of patients with stable COPD.

**Ethic and dissemination:**

This study is a systematic review and it does not involve harming to the rights of participants. Ethical approval will not be require for this study. The research results may be published in a peer-reviewed journals.

## 1 Introduction

Chronic obstructive pulmonary disease (COPD) is a respiratory system disease that is characterized by persistent respiratory tract symptoms and fixed airflow limitation.^[1]^ The main clinical symptoms are dyspnea, chronic cough with mucous production, chest tightness and wheezing.^[2–3]^ Currently, about 400 million people suffers from COPD which is the third leading cause of death in the worldwide.^[4]^ Additionally, the medical burden of COPD is significant ranking fifth in the world's economic burden of disease.^[5]^ According to statistics, the U.S. government spent nearly $50 billion on treatment of COPD in 2010.^[6]^ In China, about 1.5 million people die from COPD every year.^[7]^ COPD presents a significant challenge to the health care provider worldwide.^[8–9]^

The Global Initiative for Chronic Obstructive Lung Disease (GOLD) recommends that patients with acute exacerbation of COPD are mainly treated with drugs and oxygen therapy, while extra non-drug treatment should be used for COPD patients in stable phase, in addition to drugs.^[9]^ Non-drug treatments mainly comprised oxygen therapy and pulmonary rehabilitation training. Pulmonary rehabilitation training is widely used in COPD, with the purpose of reducing symptoms, improving quality of life, reducing medical burden, and increasing social participation. Its efficacy is widely recognized.^[1,9,11]^

Liuzijue qigong (LQG) is a traditional Chinese method of fitness based on breath pronunciation. As a part of the traditional fitness qigong series launched by Chinese Health Qigong Association, LQG performs the actions of inhaling and exhaling though different mouth patterns to control and regulate the rise and fall of the breath in the body, and completing the practice of “xu, he, hu, si, chui, xi” with breathing and pronunciation. These exercises plays a positive role in regulating respiratory system, exercise endurance and quality of life on patients with stable COPD.^[12–14]^ Despite the potential benefits of LQG for COPD management, various design and methodologic weaknesses have consistently been identified across studies. In addition, there lacks a systematic review and meta-analysis of clinical therapeutic effect about LQG on patients with COPD. So this study is a systematic review and meta-analysis of published literature on the application of LQG in patients with stable COPD, in order to provide high-quality evidence synthesis and decision basis for the rehabilitation of COPD patients.

## 2 Methods

### 2.1 Systematic review registration

This systematic review and meta-analyzes has been registered on PROSPERO (Systematic Review Registration: https://www.crd.york.ac.uk/prospero/PROSPERO registration number: CRD42020209191**).**

### 2.2 Ethics

Since this study is a systematic review and does not involve clinical trials, it does not require the approval of the Ethics Committee.

### 2.3 Search strategy

The electronic database searched were PubMed, Cochrane Library, Web of Science, China National Knowledge Infrastructure, and Chinese WanFang Data, from 1970 until September 2020. The medical subject headings (Mesh) terms were chronic obstructive pulmonary disease (COPD), liuzijue qigong. The keywords were traditional Chinese exercise; qigong; health qigong; liuzijue; Liuzijue respiratory gymnastic; randomized controlled trial; chronic obstructive pulmonary disease (COPD). A details of Search Strategies are showed on Appendix. The search strategy of this study uses a combination of Mesh terms and keywords, and is determined after repeated pre-checks, supplemented by manual search, and retrospectively included references when necessary.

### 2.4 Inclusion and exclusion criteria

#### 2.4.1 Inclusion criteria

1. Only RCTs regarding the efficacy of LQG for COPD were included.
2. Patients were diagnosed with COPD in a stable state according to the Global Initiative for Chronic obstructive pulmonary disease (GOLD), and no acute exacerbation occurred in all patients within 6 months before entering the trial.
3. The baseline interventions included routine basic treatment of western medicine, oxygen therapy, usual care, conventional respiratory therapy, health education and were equally implemented in both groups.
4. The experimental group participated in LQG, while the control group received health education, or conventional breathing exercise or or no intervention.

#### 2.4.2 Exclusion criteria

1. The papers with unscientific and unrigorous experimental design are excluded
2. The full text which could not obtained through various channels was excluded;
3. Qualitative studies, animal experiments, case reports and conference abstract reviews were excluded;
4. Documents with incomplete data or data problems were excluded;
5. Documents with inconsistent main outcome indicators were excluded;
6. Patients with COPD accompanied by other complications were excluded, these complications include bronchial asthma, bronchiectasis, bronchial tumors, tuberculosis, acute coronary syndrome, severe heart and kidney failure, poorly controlled diabetes and blood glucose, as well as patients with severe blood system diseases and mental disorders that cannot be treated with treatment;
7. Patients with COPD are in an unstable phase were excluded.

### 2.5 Data extraction and synthesis

Data were extracted independently by 2 reviewers (PG and FT) according to inclusion and exclusion criteria, and then cross-checked. If there was a dispute, it was settled through discussion. The extracted contents included:

1. The basic materials of the literature, such as the author, the year of publication, etc.;
2. Specific details of experimental design, such as randomization, allocation and hiding, blind method, basic data, intervention measures, outcome evaluation indexes, intervention time and follow-up time of the study subjects.

If the research data is found incomplete, the author was contacted by phone or email to obtain the data. If the relevant data is not obtained in the end, the article will be excluded. When RCT with multiple studies was involved, the experimental group and control group related to this study were extracted.

### 2.6 Types of outcome measures

The primary outcome included [forced expiratory volume in one second (FEV1)], [as a percentage of predicted expiratory volume in one second, FEV1 (%)], the ratio of forced expiratory volume to forced vital capacity in the first second (FEV1/FVC [%]), exercise endurance (6 minutes walking distance, [6MWD]). And the secondary outcome comprised Health-related quality of life (St. George's Respiratory Questionnaire (SGRQ) and COPD Assessment test (CAT), Dyspnea index (modified medical research council dyspnea scale, [mMRC]).

### 2.7 Literature quality evaluation

Jadad score was used to evaluate the methodological quality of each RCTs included,^[15]^ with a total score of 7 points. Scores <4 were considered as low quality studies, while scores ≥4 were considered as high quality studies.^[16]^ The risk of bias was assessed using the evaluation criteria recommended by the Cochrane Handbook 5.1.0,^[17]^ “low risk bias,” “high risk bias” and “unclear” (lack of relevant information or uncertainty of bias) were assessed for each of the included literatures. The quality evaluation of literatures were conducted independently by 2 reviewers (PG and YM). Any controversy occurring during the evaluation process were discussed with a third reviewer (WL) and resolved by consensus.

### 2.8 Statistical analysis

The RevMan5.3 and Stata14.0 software were used for meta-analysis. As a priori analysis, we also analyzed the property of data across 3 intervention durations, defined as short-term (≤3 months), medium-term (6 months), and long-term (12 months). Heterogeneity test: judged by Chi^2^ test and *I*^2^ test, if *P* < .05, *I*^2^ ≤ 50% indicate that there is homogeneity among the studies, and fixed effect model was used for analysis. If *P* ≤ .05, *I*^2^ > 50%, indicating statistically heterogeneity, random effect model was used for analysis. Finally, we used the funnel plots and Egger's regression asymmetry test to detect publication bias. To prove the reliability of our meta-analysis results, a sensitivity analysis were conducted by removing each study one by one to evaluate the consistency and quality of results. If variables in the studies included in this meta-analysis were continuous, we used the mean difference (MD) and 95% confidence interval (CI) to analyze the studies, otherwise, standardized mean difference (SMD) was used when variables were inconsistent. We considered *P* values less than .05 to be statistically significant.

## 3 Result

### 3.1 Search results

**Figure 1:**
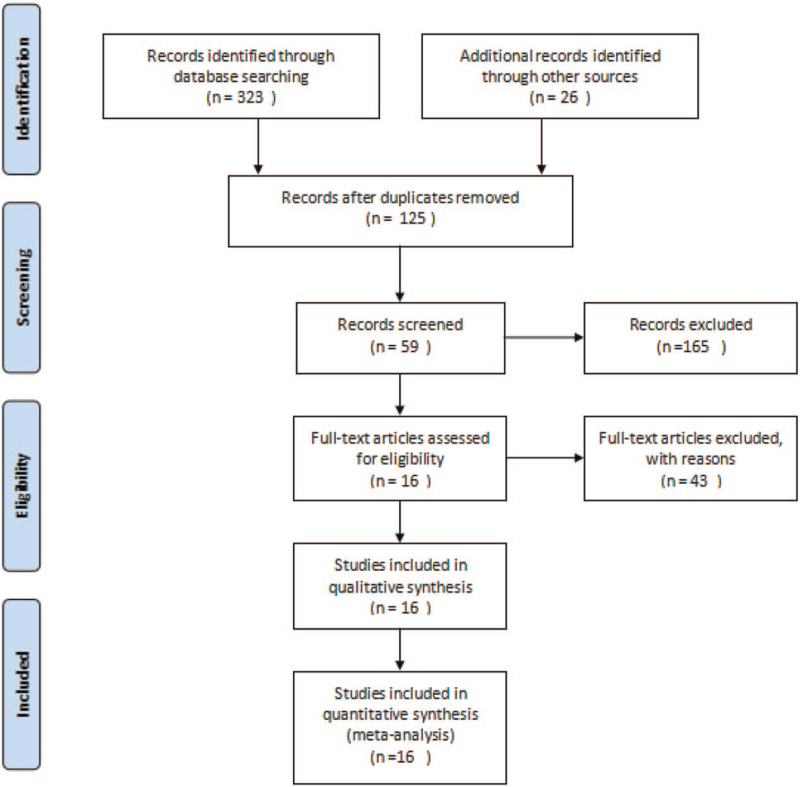
The flow diagram of the selection process.

The flow diagram of the selection process is summarized in Figure 1. Four hundred thirty nine potentially eligible reports or articles were founded through electronic searches. One hundred twenty four articles still remained by eliminating repeated records. We excluded 31 articles of these based on the title, abstract. Of the 16 remaining articles, an additional 43 were excluded. The most common reasons for exclusion were a non-RCT design, unrelated outcomes, Non-clinical research, or patients in unstable phase of COPD. Eventually, 16 RCTs were deemed eligible for inclusion and selected for the final analysis.

### 3.2 Study characteristics

A total 16 studies^[18–33]^ involving 1039 individuals were selected for this meta-analysis, 1 studies^[23]^ were published in English, and 15 studies^[18–22,24–33]^ were published in Chinese, six of which were dissertations.^[24,28–29,31–33]^ All eligible studies were published from 2008 to 2019. There were 13 studies with a sample size ≥40 participants, and the average age of participants is >60 years old. The quality of each study was assessed using the Jadad scale, 5 studies were high-quality,^[22–23,29,31,33]^ 10 studies were low-quality.^[18–21,24–28,30,32]^ The basic characteristics of the included studies were shown in Table 1.

**Table 1:** The detailed characteristics of each selected study.

					Duration of intervention					
Author, Year	Region language	Sample (L/C)	Mean age /Year (L/C)	Intervention program (L/C)	Frequency (weekly)	Time (min)	Duration (month)	Outcome	Adverse event	Jadad score
Fang DP 2012	Fuzhou, China, (Chinese)	61/60	71.75/73.10	L=Routine health advice+LQG C=Routine health advice+conventional breathing exercise	7	30	6	Dyspnea score; Exercise capacity (6-MWD)	No	3
Chen JX 2009	Fuzhou, China, (Chinese)	31/29	70.16/71.52	L=Routine health +LQG C=Routine health+ breathing exercise	7	30	3	FEV1_,_; FVC; FEV1/FVC (%); 6MWD	No	3
ZhangMM 2019	Shanghai, China, (Chinese)	67/71	71.0/70.4	L=oxygen therapy +drug+LQG C= oxygen therapy+drug	21	30	12	mMRC; AECOPD; PaCO_2_; 6MWD; SGRQ	No	2
Sun N2019	Qingdao, China, (Chinese)	56/56	65.45/64.78	L=conventional drugs + health advice+ Lip and abdominal breathing +LQG C=Conventional drugs +health advice+ Lip and abdominal breathing exercise	14	30	6	FEV1; FEV1%; 6MWD; SGRQ	No	3
Deng LJ 2018	Fuzhou, China, (Chinese)	28/26	72.37/72.60	L=Conventional drugs +LQG C=Conventional drugs + breathing exercise	5	30	3	mMRC; 6MWD; SGRQ	No	4
Wu WB 2018	Shanghai, China, (English)	16/17	67/66	L=conventional treatment+LQG C=conventional treatment+no exercise	6	40	6	FEV1_;_MMEF, FEV1%; FEV_1_/FVC (%); 6MWD; 30S ssT, repetitions; Handgrip strength; SGRQ	No	5
Jiang MN 2017	Changsha, China, (Chinese)	33/32	63.66/60.64	L=conventional treatment+LQG C=conventional treatment+breathing exercise	7	30	3	FVC, FEV1%; 6MWD; CAT	No	3
ZhangWX 2009	Fuzhou, China, (Chinese)	21/19	71.76/73.32	L=routine health+LQG C=routine health	NS	NS	3	6MWD	No	2
Zhu Z 2011	Nanjing, China, (Chinese)	20/22	60.85/60.85	L=conventional treatment+LQG C=conventional treatment	NS	NS	3	FEV1_;_ FEV1%; FEV1/FVC (%)	No	2
Chen JX 2008	Fuzhou, China, (Chinese)	21/19	71.76/73.32	L=Routine health+LQG C=Routine health	NS	NS	3	MRC; FEV1_;_ FEV1%; FEV1/FVC (%)	No	3
Li DX 2011	Fuzhou, China, (Chinese)	30/30	72.77/70.13	L=conventional treatment+health advice+LQG C=conventional treatment+health advice+breathing exercise	7	30	3	FEV1; FEV1%; FEV1/FVC (%) Raw;sGaw;MIP;MEP	No	3
Chen FX 2015	Fuzhou, China, (Chinese)	30/32	76.53/76.59	L=Conventional treatment+health advice+LQG C=conventional treatment+health advice	5∼7	30	3	FEV1; FEV1%; FEV1/FVC (%); IL-8, TNF-α, Fn	No	4
Lan Y 2016	Luzhou, China, (Chinese)	42/42	67.24/67.02	L=drug+LQG C=drug	10	60	3	FEV1%;FEV1/FVC (%); CAT;	No	2
He JF 2019	Beijing, China, (Chinese)	30/30	66.26/63.20	L=Conventional treatment+health advice+LQG C=conventional treatment+health advice	14	10	6	AECOPD; CAT; FEV1%;FVC FEV1/FVC (%); T lymphocyte subsets	No	5
Li R 2018	Beijing, China, (Chinese)	15/15	67.73/67.21	L=conventional treatment+LQG C=conventional treatment+no exercise intervention	3	60	3	FEV1%; FVC; CAT, FEV1/FVC (%);	No	3
Wang LB 2015	Shanghai, China, (Chinese)	17/19	66.06/65.74	L=conventional treatment+LQG C=conventional treatment	6	20	6	BMI, 6MWD; mMRC; FEV1% FEV1/FVC (%);SGRQ	No	6

### 3.3 Methodological quality assessment

**Figure 2:**
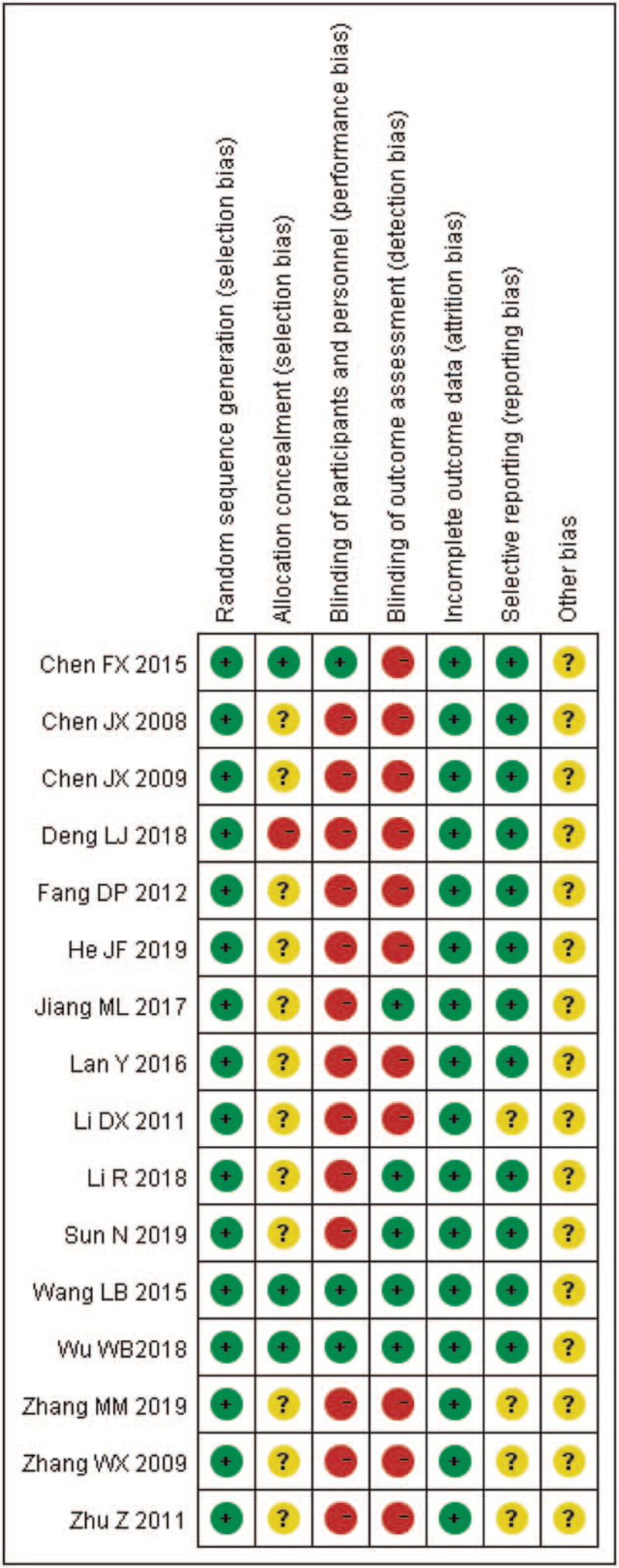
Risk of bias summary: Review authors’ judgments of bias items for each included study.

The methodological quality of all included studies were evaluated according to the bias risk assessment tools provided by the Cochrane Collaboration. All of the included trials described randomized allocation, and they were low risk in the fields of randomized allocation. Twelve studies were classified as having an unclear risk in the fields of allocation concealment^[18–21,24–28,30–32]^ and one was high risk.^[22]^ There was high risk of bias in the domain of blinding of participants and personnel, only 3 studies^[23,29,33]^ used single-blind method, but no specific method of blinding was mentioned in these studies. Only five of them were shown to blind their outcome assessment.^[21,23–24,32–33]^ All trials reported methods with a low risk of incomplete outcome data and 38 studies were at low risk of bias. With regard to selective outcome reporting bias, 10 studies were determined as low risk and the remaining were determined as unclear risk. All studies were graded as unclear risk of other bias. These results were summarized in Figure 2.

### 3.4 Effects of liuzijue qigong on primary outcomes

#### 3.4.1 Effects of liuzijue qigong on pulmonary function

**Figure 3:**
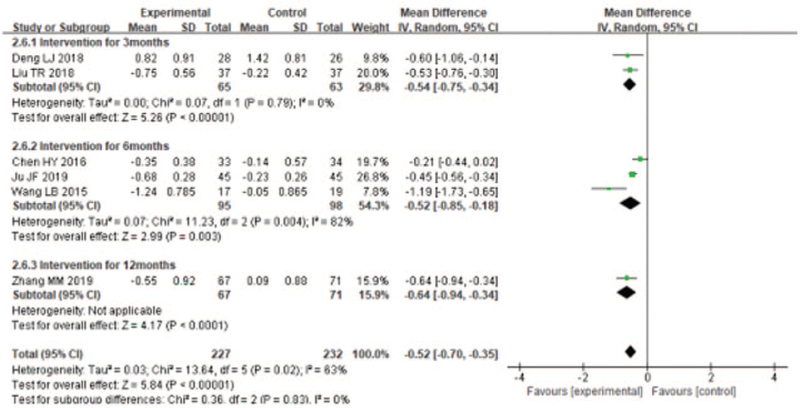
Meta-analyzes of the effect of liuzijue health qigong on mMRC compared with the control group as conducted in different intervention periods.

**Figure 4:**
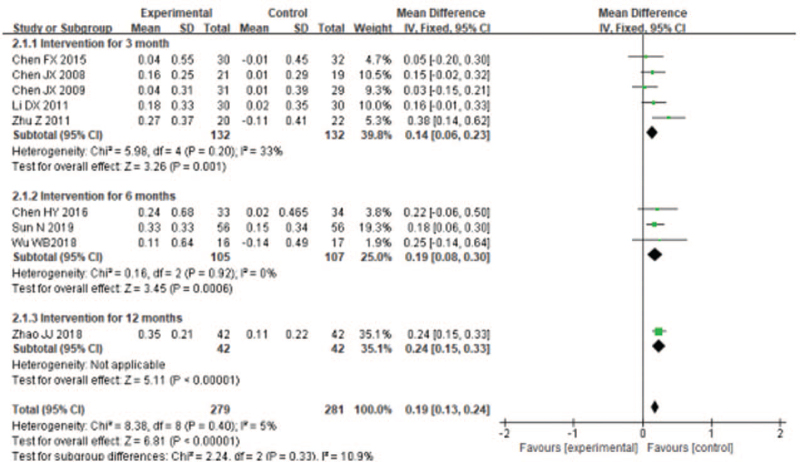
Meta-analyzes of the effect of liuzijue health qigong on FEV_1_ (L) compared with the control group as conducted in different intervention periods.

**Figure 5:**
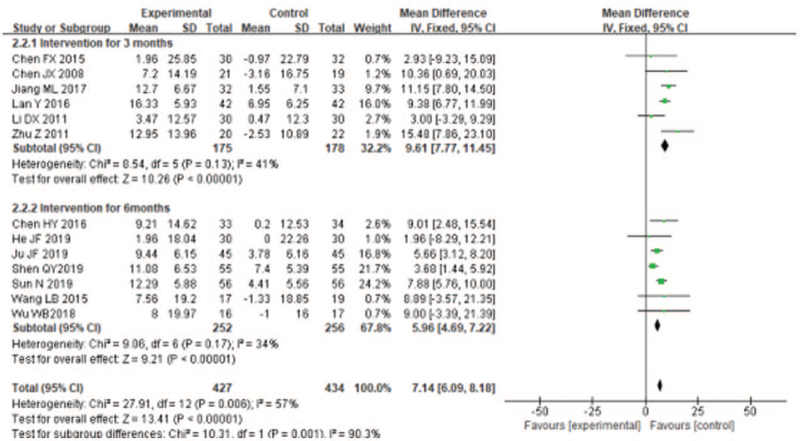
Meta-analyzes of the effect of liuzijue health qigong on FEV_1_ (%) compared with the control group as conducted in different intervention periods.

Pulmonary function test was the main objective index to judge airflow limitation. FEV1, FEV1%, and FEV1/FVC (%) are important component of pulmonary function. Seven trials^[19,21,23,26–29]^ used FEV1 to evaluate the therapeutic effect of LQG on stable COPD patients. The fixed effects model was utilized to integrate the results (*I*^2^ = 7%). The results showed that, overall, LQG significantly improved the FEV1 compared with the control group (MD = −0.16, 95% CI [0.09, 0.23], *P* < .00001; Fig. 3), the efficacy on FEV1 were noticeable in both short term duration of study (MD = 0.14, 95% CI [0.06, 0.23], *P* = .001; Fig. 3] and medium-term duration of study (MD = 0.19, 95% CI [0.07, 0.30], *P* = .002; Fig. 3). Eleven studies^[21,23–24,26–33]^ used FEV1% to assess the improvement of pulmonary function of COPD patients by LQG. The fixed effects model was conducted to incorporate the results, they had high heterogeneity (*I*^2^ = 63%). The effect size of the studies^[21,23–24,26–33]^ showed that Liuzijue exercise could significantly improve the FEV1% (MD = 9.71, 95% CI [8.44, 10.98], *P* < .00001; Fig. 4). The treatment effect on FEV1% were prominent in both short term (MD = 11.02, 95% CI [9.39, 12.66], *P* < .00001; Fig. 4) and medium-term duration of study (MD = 7.71, 95% CI [5.69, 9.73], *P* < .0001; Fig. 4). Nine trials used FEV1/FVC (%) to evaluate the enhancement of lung function of COPD patients. The fixed effects model was conducted to incorporate the results (*I*^2^ = 50%). The effect size of the studies^[19,23,26–29,31–33]^ showed that Liuzijue breathing exercise could significantly improve the FEV1/FVC (%) (MD = 4.81, 95%CI [2.12, 7.51], *P* = .0005; Fig. 5). The effect of FEV1/FVC (%) had significant improvement for COPD in short term (MD = 5.35, 95% CI [1.93, 8.78], *P* = .002; Fig. 5) and with insignificant in medium-term duration of study (MD = 2.99, 95% CI [−1.11, 7.09], *P* = .15; Fig. 5).

#### 3.4.2 Effects of liuzijue qigong on exercise endurance

**Figure 6:**
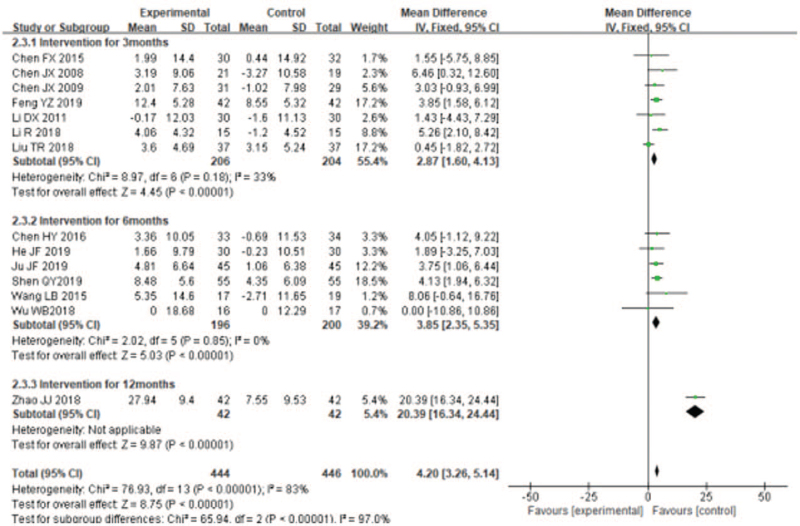
Meta-analyzes of the effect of liuzijue health qigong on FEV_1_/FVC (%) compared with the control group as conducted in different intervention periods.

Nine studies used 6MWD to evaluate the effect of LQG on exercise endurance of COPD patients. The random effects model was adopted to incorporate the results (*I*^2^ = 43%). The overall effect of the studies^[18–25,33]^ found that LQG was associated with significantly improvement the 6MWD compared with the control group (MD = 21.89, 95% CI [14.67, 29.11], *P* < .00001, Fig. 6), The effect on 6MWD were remarkable in both short term duration of study (MD = 16.78, 95% CI [14.18, 19.38], *P* < .0001, Fig. 6) and medium-term duration of study (MD = 42.72, 95% CI [28.36, 57.08], *P* < .00001, Fig. 6), but there was no significant effect in the studies with a long-term duration of study (MD = 23.11, 95% CI [−3.35, 49.57], *P* = .09; Fig. 6].

### 3.5 Effects of liuzijue qigong on secondary outcomes

#### 3.5.1 Effects of liuzijue qigong on health-related quality of life

**Figure 7:**
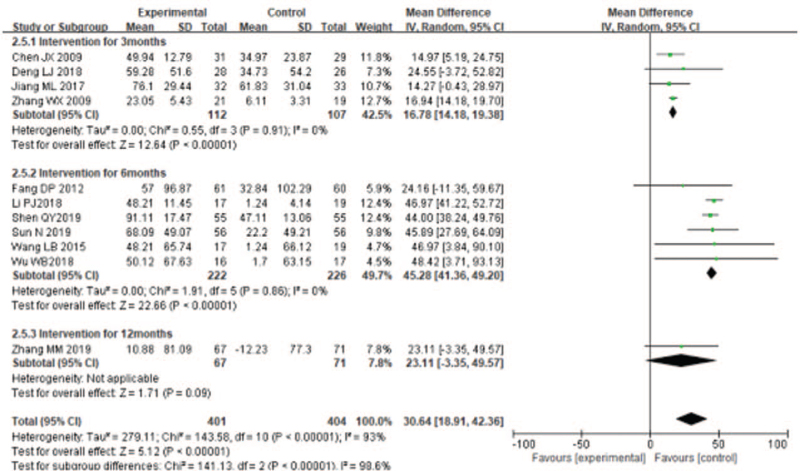
Meta-analyzes of the effect of liuzijue health qigong on 6MWD compared with the control group as conducted in different intervention periods.

Five of 7 trials used CAT to evaluate the effect of LQG on health-related quality of life of COPD patients while the other 2 articles used SGRQ. There was high heterogeneity among the included studies (*I*^2^ = 65%), and a random effect model was used for merge the results. The overall effect of the studies^[20–24,29–31,33]^ showed significantly improvement the health-related quality of life compared with the control group (SMD = −0.84, 95% CI [−1.12, −0.55], *P* < .00001, Fig. 7), The effect on health-related quality were outstanding in short term duration of study (SMD = −0.70, 95% CI [−1.01, −0.38], *P* < .001, Fig. 7), medium-term duration of study (SMD = −1.10,95%CI [−1.67, −0.52], *P* = .0002, Fig. 7), and long-term duration of study (SMD = −0.57, 95% CI [−0.91, −0.23], *P* = .001; Fig. 7].

#### 3.5.2 Effects of liuzijue qigong on dyspnea

**Figure 8:**
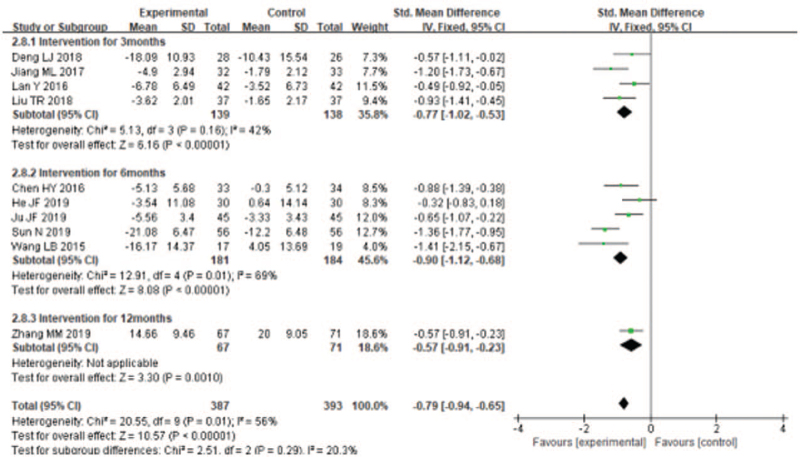
Meta-analyses of the effect of liuzijue health qigong on Health-related quality (CAT, SGRQ) compared with the control group as conducted in different intervention periods.

Only 3 studies^[20,22,33]^ used the mMRC to evaluate dyspnea of patients with COPD. The random effects analysis was managed to merge the results (*I*^2^ = 42%). The results showed that LQG significantly lowered the mMRC compared with the control group (MD = −0.73, 95% CI [−0.96, −0.50], *P* < .00001, Fig. 8].

### 3.6 Adverse events

None of the articles informed adverse events. Thence, this information could not be searched from the RCTs analyzed.

### 3.7 Sensitivity analysis

**Figure 9:**
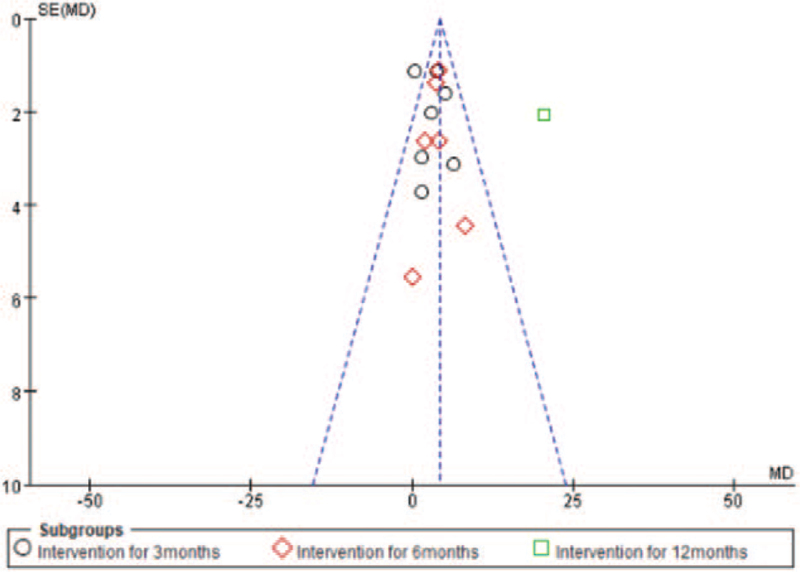
Funnel plot for evaluating the publication bias.

Some results of this study had high heterogeneity for example, FEV1 (%), FEV1/FVC (%) and health-related quality, by removing single studies for example the study^[26]^ of FEV1 (%), the study^[32]^ of FEV1/FVC (%), study^[31]^ of health-related quality, the sensitivity analyses showed obvious changes in the statistical significance of outcomes Fig. 9.

### 3.8 Publication bias

As shown from the Egger asymmetry tests, there was little indication of publication bias on the these outcomes (*P* = .764 for FEV1; *P* = .436 for FEV1%; *P* = .076 for 6MWD; *P* = .348 for health-related quality, respectively), only the outcome of FEV1/FVC% (*P* = .048) was showed publication bias.

## 4 Discussion

To the best of our knowledge, this is the first systematic review with meta-analytical method to objectively evaluate the therapeutic effects of LQG for stable COPD patients. All eligible RCTs were published between 2008 and 2019, indicating that it is a newly broadening research hotspot. The purpose of this current review was to evaluate the efficacy and safety of LQG for COPD patients. No adverse events were found in any of these studies, and the positive findings in the current review are in line with previous meta-analyzes of randomized controlled trials investigating the beneficial effects of other Chinese traditional exercises (e.g., Tai Chi, Baduanjin and Meditative movement) for COPD patients.^[34–36]^

In our study, we created subgroups based on the different intervention duration time of LQG to evaluate the overall effect and each subgroup's effect, which would informed COPD patients of the effect of different intervention duration times. Generally, compared with conventional therapy, our study found that LQG was helpful for COPD rehabilitation as assessed by FEVI, FEV1 (%), FEV1/FVC (%), 6MWD, mMRC, and health-related quality.

6MWD has been used as a simple and valid evaluation parameter for exercise tolerance of COPD patients.^[37–39]^ In our study, we found the distance of the 6MWD in the LQG group was increased compared with the distance in the control group. In subgroup analysis, whether it was a short-term intervention or a medium-term intervention, LQG had a positive effect for improving 6MWD of COPD patients. However, long-term intervention has no obvious effect, the main reason is that there is only 1 literature included. Declining of exercise endurance and lung function are the main characteristics of COPD, and there are numerous reasons for the decreasing of patients’ exercise ability. It is generally believed that the patient's movement is restricted to airflow obstruction, lung hyperinflation and gas exchange barriers during the activity process,^[40–41]^ furthermore, the movement restriction makes the patient unable to engage in related sports which causes the patient's exercise ability to further decline,^[42]^ then this will form a vicious circle. LQG contains breathing exercises and limb movement, which is not only beneficial to the flexibility and functional coordination of the upper limb muscles, but it also strengthens the function of the lower limbs. Thus LQG could prolong the 6MWD of COPD patients.

FEV1, FEV1%, FEV1/FVC (%) are important indicators to reflect the degree of airway ventilation and obstruction of COPD patients, which can assess the severity of the patient's clinical symptoms and disease severity.^[43]^ In our study, LQG improved patients’ pulmonary function [i.e., FEV1, FEV1 (%), FEV1/FVC (%)] compared with control group by the pool effect of our meta-analysis. In subgroup analysis, LQG had a good effect on these index of lung ventilation function [FEV1, FEV1 (%), FEV1/FVC (%)] in COPD patients regarding short-term or mid-term duration intervention. This finding may be because LQG can enhance the strength of respiratory muscle,^[44]^ Also “Xu, Si and He” word tactic of LQG can extend expiratory time, improve the airway pressure, avoid premature closure of the airway, increase pulmonary ventilation function in patients with COPD.^[45]^ However, our finding is contrary to Tong HX's^[46]^ research viewpoint that the Liuzijue of traditional health qigong cannot improve the lung function of COPD patients. For this reason, we carefully read Tong's study of meta-analysis and found that there is only 1 article about Liuzijue's intervention in COPD patients included in his study of meta-analysis, which is not sufficient to demonstrate the effectiveness of LQG intervention in COPD patients. In the actually practice of LQG, the breathing method is beneficial to improve the abdominal muscle tension, increase movement range of the diaphragm rise and fall, enhance the strength of respiratory muscle, and thus obtain the greatest improvement of lung function. So patients with COPD could choose LQG for improving respiratory function.

Currently, improving COPD patients’ dyspnea and enhancing their exercise endurance were the main target through LQG. Declining of exercise endurance and respiratory function directly affects the quality of life of COPD patients. Therefore, the assessment of the health-related quality of life of patients should be an important part of the effect of treating patients. CAT respiratory questionnaire and the SGRQ respiratory questionnaire were widely used for assessing the quality of life of patients with COPD. In our study, the overall effect size showed that LQG significantly improved the health-related quality of life compared with the control group. Additional, good evidence was found in our study that LQG decreased mMRC score of dyspnea. The underlying mechanism is that LQG has a positive effect on the T cell immune function of patients with stable COPD and prevent patients from getting sick more easily,^[31]^ As a form of traditional fitness exercise, limb training of LQG can effectively relieve dyspnea symptoms during activities while adjusting the respiratory function and relaxing the whole body function, so it has better effects on health-related quality of life of COPD patients.^[47–48]^

In subgroup analysis of our study, LQG also had better evidence of the effect on FEV1, FEV1 (%), FEV1/FVC (%), 6MWD and health-related quality of life in both short term and medium-term duration of study. However, the long-term intervention duration of LQG had no effect, and it may be that there is too little literature to prove the evidence. From our study, we suggested that the intervention duration of LQG lasts at least 3 to 6 months.

### 4.1 Limitation

Athough we have comprehensive analysis and assessed all eligible studies, it still has some limitation. First, 15 of 16 RCTs in this meta-analysis were published in Chinese and little of relevant foreign RCTs, there may be publication bias that the result of this study were regional. Secondly, in our study, some results had high heterogeneity with regards to intervention intensity, duration, and frequency that may have contributed to unwanted heterogeneity and may have further influenced the outcomes. Even though our classification of intervention durations (i.e., short, medium and long term), the relatively small number of studies included in each category did not allow us to effectively explanation the heterogeneity underlying the different studies in our random effect models. Thirdly, most of the studies showed only the randomized trials, but no specific methods of random sequence generation, RCTs of allocation concealment, and blinding of outcome assessment. There were only 3 studies which reported single blinding. The methodological quality of many of the included RCTs was generally low and might have a high risk of bias.

### 4.2 Practical implications

First, research design should be carried out with stable COPD patients as the inclusion objects, high-quality, large sample RCT. Secondly, LQG is a kind of aerobic exercise with medium and low intensity,^[43,49–50]^ so we should try to improve the exercise intensity of the traditional fitness method in the research. Thirdly, the mechanism of COPD is still under further exploration at the present, so it is necessary to add some other index to comprehensively evaluate the efficacy of COPD patients, such as acute exacerbation of COPD, BODE index, peripheral muscle strength and cellular immune factors.

## 5 Conclusion

In summary, this meta-analysis of RCTs suggested that LQG had positive effects in field of lung function, exercise endurance, health-related quality of life and dyspnea of patients with stable COPD.

## Author contributions

**Data curation:** Pincao Gao, Fang Tang.

**Formal analysis:** Kai He.

**Methodology:** Pincao Gao, Weiguo Liu, Yu Mo.

**Project administration:** Kai He.

**Software:** Pincao Gao, Kai He, Yu Mo.

**Supervision:** Fang Tang, Weiguo Liu.

**Writing – original draft:** Pincao Gao.

**Writing – review & editing:** Pincao Gao, Fang Tang, Kai He.
